# The short-term impact of ambient air pollution on years of life lost from cancer in Pudong New Area, Shanghai: A time series study

**DOI:** 10.1371/journal.pone.0360049

**Published:** 2026-10-27

**Authors:** Xing Zhong, Jiayi Sheng, Yichen Chen, Hua Chen, Siyue Han, Xiaobin Qu, Lianghong Sun, Sen Wang, Caixia Hu, Lipeng Hao

**Affiliations:** 1 School of Public Health, Fudan University, Shanghai, China; 2 Shanghai Pudong New Area Centre for Disease Control and Prevention (Shanghai Pudong New Area Health Supervision Institute), Shanghai, China; National Center for Chronic and Noncommunicable Disease Control and Prevention, Chinese Center for Disease Control and Prevention, CHINA

## Abstract

**Objectives:**

Previous studies have shown that ambient air pollution was associated with an increased risk of cancer mortality. However, the mortality outcome treated every death equally and ignored the significance of premature death.

**Methods:**

We conducted a time series study to evaluate the short-term impact of ambient air pollution on years of life lost (YLLs) from cancer in Pudong New Area, Shanghai. Data on air pollutants, meteorological factors, cancer deaths, and years of life lost were collected from 2013 to 2021. In this study, the distributed lag nonlinear model (DLNM) combined with linear (for YLLs) and quasi-Poisson (for mortality) regression models was used to investigate the lag-specific association between air pollution exposure and two outcome indicators of cancer. A stratified analysis was performed according to sex, age, and education.

**Results:**

For YLLs, per 10 μg/m^3^ increase in PM_2.5_, PM_10_, SO_2_, NO_2_, and O_3_ exposure was significantly associated with YLL increases of 2.22 (95% CI: 0.71, 3.74), 1.04 (95% CI: 0.17, 1.91), 5.05 (95% CI: 0.74, 9.36), 1.88 (95% CI: 0.09, 3.66), and 0.96 (95% CI: 0.14, 1.78) years on lag0-1, respectively. Marginal differences in impact estimates were observed among men, younger populations, and those with higher education. In addition, the difference in the impact of O_3_ exposure on cancer mortality among low- and high-education subgroups was statistically significant.

**Conclusions:**

Short-term exposure to ambient air pollution was positively associated with increases in YLLs and cancer mortality in Pudong New Area, Shanghai.

## Introduction

Ambient air pollution remains one of the most significant environmental threats to human health worldwide. Driven by industrialization, urbanization, fossil fuel combustion, and agricultural activities [1], ambient air pollution is becoming increasingly severe, mainly including fine particulate matter (PM_2.5_), inhalable particulate matter (PM_10_), sulfur dioxide (SO_2_), nitrogen dioxide (NO_2_), ozone (O_3_), and carbon monoxide (CO). According to the World Health Organization, 99% of the global population breathes air exceeding WHO air quality guidelines, with low- and middle-income countries bearing the highest exposure burdens. The persistence of those pollutants in the atmosphere creates scenarios of chronic and short-term exposure, transforming air quality into a critical determinant of population health. Due to the diverse physical and chemical properties of air pollutants, air pollution exposure has harmful effects on the respiratory system, cardiovascular system, ophthalmic system, dermatological system, neuropsychiatric system, hematological system, immune system, and reproductive system [2].

Cancer is a typical chronic noncommunicable disease, characterized by the uncontrolled growth and spread of abnormal cells. These cells can destroy healthy tissues, form tumors, and disrupt bodily functions [3]. Cancer is the second leading cause of death worldwide, accounting for one-sixth of all deaths in 2020 (approximately 10 million deaths). The most common cancers are breast cancer, lung cancer, colon cancer, rectal cancer, and prostate cancer. In China, the burden of cancer remains relatively heavy. In 2022, the estimated number of cancer deaths was 2.57 million, with 1.63 million among men and 0.94 million among women. Lung cancer was the leading cause of cancer-related deaths, followed by liver cancer, stomach cancer, and colorectal cancer. In 2022, approximately 730,000 deaths were attributed to lung cancer, accounting for 28.5% of all cancer-related deaths [4].

Air pollution is also one of the major risk factors for cancer. A large cohort study in America has revealed that the risk of all cancer mortality was adversely associated with each 10 µg/m^3^ increase in PM_2.5_ concentration in the full cohort (hazard ratio: 1.15, 95% CI: 1.08 to 1.22) [5]. A machine learning analysis of 35 environmental sources of pollution in Italy has proved that air quality ranked first for importance concerning the average cancer mortality rate [6]. An ecological study in Hangzhou, China, has shown that a 10 µg/m^3^ increase in pollutant (PM_2.5_, PM_10_, NO_2_, and SO_2_) concentration was associated with increased risk of cancer death in females [7]. A meta-analysis of seven observational studies has investigated the association between PM_2.5_ exposure (per 10 µg/m^3^ increase) and increased relative risk of colorectal cancer (RR: 1.42, 95% CI: 1.12 to 1.79, P = 0.004) [8]. One study from Osaka, Japan, has found that an interquartile range increase in concentration of PM_2.5_, NO_2_, SO_2_, and CO was positively associated with increased risk of lung cancer mortality [9]. Another study from California has also found that exposure to NO_2_ and PM_2.5_ may adversely impact ovarian cancer-specific survival [10].

However, past studies have chosen mortality as the outcome measure to explore the association between air pollution exposure and cancer mortality risk, ignoring the importance of premature death [9,11]. In this study, YLL was selected as a supplementary outcome measure for mortality, accounting for life expectancy at death and assigning greater weight to deaths among younger people. YLL can better reveal health inequities, identify vulnerable populations, and assist public health departments or policymakers in prioritizing interventions and resource allocation in these areas.

Herein, the distributed lag nonlinear model combined with linear (for YLLs) and quasi-Poisson (for mortality) regression models was used to investigate the lag-specific association between air pollution exposure and two outcome indicators of cancer. A stratified analysis was also conducted according to sex, age, and education. The objective of this study was to evaluate the short-term impact of ambient air pollution on YLLs from cancer in Pudong New Area, Shanghai.

## Materials and methods

### Air pollution and meteorological data

Daily average concentration data from January 1, 2013, to December 31, 2021, for fine particulate matter (PM_2.5_), inhalable particulate matter (PM_10_), sulfur dioxide (SO_2_), nitrogen dioxide (NO_2_), ozone (O_3_), and carbon monoxide (CO) were collected from the monitoring station at Shanghai Pudong International Airport. The monitoring station is located in Zhuqiao Town, Pudong New Area, geographically situated exactly in the middle of the district’s northern and southern sections. The meteorological data, including daily average temperature and relative humidity, were obtained from the Pudong New Area Meteorological Bureau, which reflected the meteorological characteristics of the Pudong New Area during the same period.

### Mortality and YLL data

Daily mortality data in Pudong New Area from 2013 to 2021 were derived from the vital statistics database. Using the Mortality Registration System of Pudong New Area, which compiles data from medical institutions of all levels and the local civil administration department, the mortality data were checked monthly against the local population registry. The personnel of Pudong New Area Centre for Disease Control and Prevention (PDCDC) performed periodic assessment and data cleaning according to standard guidelines. Cause of death was coded according to the International Classification of Diseases, 10th revision (ICD-10), and deaths due to malignant tumor (ICD-10: C00-C97) were classified by underlying cause. The direct and underlying causes of death were coded by qualified staff, and all these records were further examined and verified by the PDCDC. After matching for age and sex, we calculated the YLL values by using the life table of Pudong New Area (S1 and S2 Tables) for each cancer-related death and then summed these values to obtain daily YLLs attributable to cancer. Both YLLs and deaths due to cancer, stratified by sex (male and female), age (<75 years and ≥75 years), and education level (illiterate and elementary school education, higher than middle school education), were used as the analysis indicators. The study design was in accordance with the ethical guidelines of the 1975 Declaration of Helsinki and has been approved by the Medical Ethics Committee of Shanghai Pudong New Area Center for Disease Control and Prevention (PDCDCLL-20230605–001).

### Statistical analysis

For descriptive statistics, the general distribution of air pollution, meteorological variables, YLLs, and daily cancer deaths in Pudong New Area from 2013 to 2021 was described using the mean, standard deviation, minimum value, 25th percentile, median, 75th percentile, and maximum value.

Firstly, we performed a time series study with DLNM based on a generalized linear model to assess the impact of ambient air pollution (PM_2.5_, PM_10_, SO_2_, NO_2_, O3, and CO) on daily YLLs and mortality from cancer. DLNM is a model that captures the nonlinear effects of exposure variables and complex dependencies in the lag time dimension by constructing cross-lagged basis functions [12]. It integrates the instantaneous effects (spatial dimension) and lagged effects (time dimension) of exposure variables (such as pollutant concentration) into a unified mathematical framework, thereby quantifying the nonlinear effects of exposure variables on health outcomes in different time windows [13]. As daily cancer death counts represent rare events, a quasi-Poisson regression with a log link function was used for the DLNM model. Natural cubic splines (ns) were used to control for potential confounders, including secular trend, seasonal variations, relative humidity, and temperature within the model framework. The day of the week (DOW) effect was controlled using indicator variables. The model is as follows:


$$\begin{array}{c} Log\left[E(Yt)\right]=\alpha +\beta Z(t,l)+ns(time,df)+ns(rh,df)+ns(temp,df)+DOW \end{array}$$

The daily YLLs due to cancer in Pudong New Area were found to approximately follow a normal distribution (S1 Fig). The model is as follows:


$$\begin{array}{c} YLLt=\alpha +\beta Z(t,l)+ns(time,df)+ns(rh,df)+ns(temp,df)+DOW \end{array}$$

where *E*(*Yt*) represents the daily count of cancer deaths on day t; *YLLt* is the sum of YLL caused by cancers that occurred on day *t*; *α* is the intercept; and *Z*(*t*,*l*) is the cross-basis object we used to assess the impact of air pollution exposure, in which *Z* is the daily mean concentrations of air pollutants at day *t* and *l* is the lag days. *β* is the coefficient for *Z*(*t*,*l*); *ns* is the natural cubic spline function; *temp* is the daily mean temperature; *rh* is the daily average relative humidity; *DOW* is a categorical variable for day of the week (used to control for day-of-week effects); and df is the degree of freedom. Referring to previous studies [14,15], we set the degrees of freedom for daily mean temperature and relative humidity to 6 and 3, respectively. According to the Akaike Information Criterion (AIC), the df for the time variable was set to 2 per year. The df for lag structures in pollutant cross-basis were set to 2. Finally, we set a maximum lag of 7 days to explore the lag pattern of air pollution [16,17].

DLNM was conducted to explore the lag-specific association between air pollution exposure and daily YLLs and mortality from cancer. The relative risk (RR) and excess risk (ER%) of cancer mortality, as well as the increase in cancer-related YLLs, were calculated for each 10 µg/m^3^ increase (1 mg/m^3^ for CO) in air pollutant concentrations. The stability of these associations was examined by using two-pollutant models with another air pollutant in the same period. A stratified analysis was also conducted according to sex, age, and education level. The implementation of the two-pollutant model and stratified analysis was based on the lag0-1 period, considering that the significant lag effects of air pollutants were generally observed on lag0-1 [18]. We calculated the 95% confidence interval (95% CI) to assess the statistical significance of differences between the subgroups (e.g., male and female subgroups). The 95% CI was calculated as follows:


$$\begin{array}{c} {\text{Q}}_{\text{1}}-{\text{Q}}_{\text{2}}\pm \text{1}.\text{96}\sqrt{{\text{SE}}_{\text{1}}+{\text{SE}}_{\text{2}}} \end{array}$$

where Q_1_ and Q_2_ are the estimates of the two subgroups, and SE_1_ and SE_2_ are the standard errors of these subgroups [19].

### Sensitive analysis

We conducted several sensitivity analyses to ensure reliability of the research results. First, we adjusted the parameters in the model to ensure the robustness of the DLNM. The degrees of freedom for temperature and relative humidity were set to 4. In terms of controlling secular trends, the degree of freedom for the time variable was set from 1 to 3 per year. Second, we excluded the study data from January 1, 2020, to December 31, 2021, to observe the impact of the COVID-19 pandemic on our results. The results of the sensitivity analysis showed no significant changes, indicating that our findings are relatively reliable (S3–S8 Tables).

In this study, all statistical analyses were conducted with R software (version 4.5.0). The “dlnm” package and “gnm” package were used to construct the distributed lag nonlinear model (DLNM) model. *P* < 0.05 was considered to be statistically significant.

## Results

### Descriptive statistics

Table 1 lists basic information on air pollutants, meteorological variables, YLLs, and daily cancer deaths in Pudong New Area during 2013–2021. The average daily concentrations of PM_10_, PM_2.5_, SO_2_, NO_2_, O_3,_ and CO were 57.59 μg/m^3^, 31.11 μg/m^3^, 12.71 μg/m^3^, 37.03 μg/m^3^, 96.52 μg/m^3,^ and 0.68 mg/m^3^, respectively, during 2013 to 2021 in Pudong New Area, Shanghai (Table 1). The mean temperature was 17.54°C, and the mean relative humidity was 75.46%. We identified a total of 65002 cancer deaths from 2013 to 2021. The mean daily death count and YLLs due to cancer were 19.76 and 357.50, respectively. The average daily death count and YLL for males, younger people, and populations with a higher education level were higher than those of females, older patients, and those with lower education levels, respectively.

**Table 1:** Descriptive statistics of air pollutants, meteorological factors, YLLs, and mortality data in Pudong New Area, 2013-2021.

Variables	Mean	Standard deviation	Minimum	25th percentile	Median	75th percentile	Maximum
PM_2.5_ (μg/m^3^)	31.11	22.19	3.83	16.33	24.83	38.46	251.71
PM_10_ (μg/m^3^)	57.59	38.73	6.93	32.71	46.71	70.72	542.46
SO_2_ (μg/m^3^)	12.71	9.35	2.31	6.98	10.31	14.77	99.73
NO_2_ (μg/m^3^)	37.03	18.47	2.67	23.72	33.05	46.54	139.51
O_3_ (μg/m^3^)	96.52	44.08	5.54	64.75	91.06	120.90	284.15
CO (mg/m^3^)	0.68	0.27	0.21	0.50	0.62	0.80	3.67
Temperature (°C)	17.54	8.43	−5.40	10.25	18.20	24.40	34.60
Relative humidity (%)	75.46	12.98	22.60	67.50	77.00	85.00	100.00
**Years of life lost**							
Total	357.50	94.30	116.50	293.30	349.50	417.80	706.10
Male	213.29	69.50	22.23	164.31	208.27	256.83	489.53
Female	144.21	62.65	0.00	98.71	139.21	182.43	401.35
Age < 75 years	267.10	87.73	19.30	205.80	260.50	322.20	611.50
Age ≥ 75 years	90.35	31.30	9.11	68.35	88.71	110.60	251.21
Low education	136.57	52.88	11.32	98.64	130.61	170.19	355.56
High education	220.93	77.03	9.38	165.67	214.97	271.24	492.00
**Daily death counts**							
Total	19.76	4.62	7.00	17.00	20.00	23.00	40.00
Male	12.23	3.57	2.00	10.00	12.00	15.00	27.00
Female	7.53	2.83	0.00	5.00	7.00	9.00	17.00
Age < 75 years	10.70	3.32	1.00	8.00	10.00	13.00	24.00
Age ≥ 75 years	9.07	3.05	1.00	7.00	9.00	11.00	24.00
Low education	9.24	3.13	1.00	7.00	9.00	11.00	21.00
High education	10.52	3.40	1.00	8.00	10.00	13.00	23.00

Table 2 shows the Spearman correlations between daily meteorological variables and air pollutants in Pudong New Area, Shanghai. The correlations among different air pollutants were mostly positive, while the relationships between pollutants and meteorological factors (temperature and relative humidity) were predominantly negative.

**Table 2:** Spearman correlation between air pollutants and meteorological factors.

	PM_2.5_	PM_10_	SO_2_	NO_2_	O_3_	CO	Temperature	Relative humidity
PM_2.5_	1	**0.881**	**0.676**	**0.642**	**0.138**	**0.806**	**−0.294**	**−0.249**
PM_10_		1	**0.679**	**0.581**	**0.221**	**0.678**	**−0.253**	**−0.437**
SO_2_			1	**0.404**	**0.077**	**0.558**	**−0.191**	**−0.327**
NO_2_				1	**−0.099**	**0.647**	**−0.425**	**−0.133**
O_3_					1	−0.004	**0.415**	**−0.287**
CO						1	**−0.238**	**−0.099**
Temperature							1	**0.262**
Relative humidity								1

The bold number indicates the *P* < 0.05.

### Associations between air pollutants and cancer outcomes

Figs 1 and 2 show the lag-specific associations between each 10 μg/m^3^ increase in air pollution exposure (1 mg/m^3^ for CO) and the estimated increases of YLLs and RR on different lag days. Due to air pollution exposure, the nonlinear lag patterns of YLLs were similar to those of cancer mortality. For the impact of single-day lag, there were apparent decreasing trends in estimated increments of YLLs and RR from lag0 to lag5. The largest and significant estimates were observed on lag0. For moving average concentrations, the maximum estimates with the narrowest confidence intervals mostly occurred on lag0-1. In addition, the estimated increases of YLLs and RR due to CO exposure were not significant.

**Fig 1:**
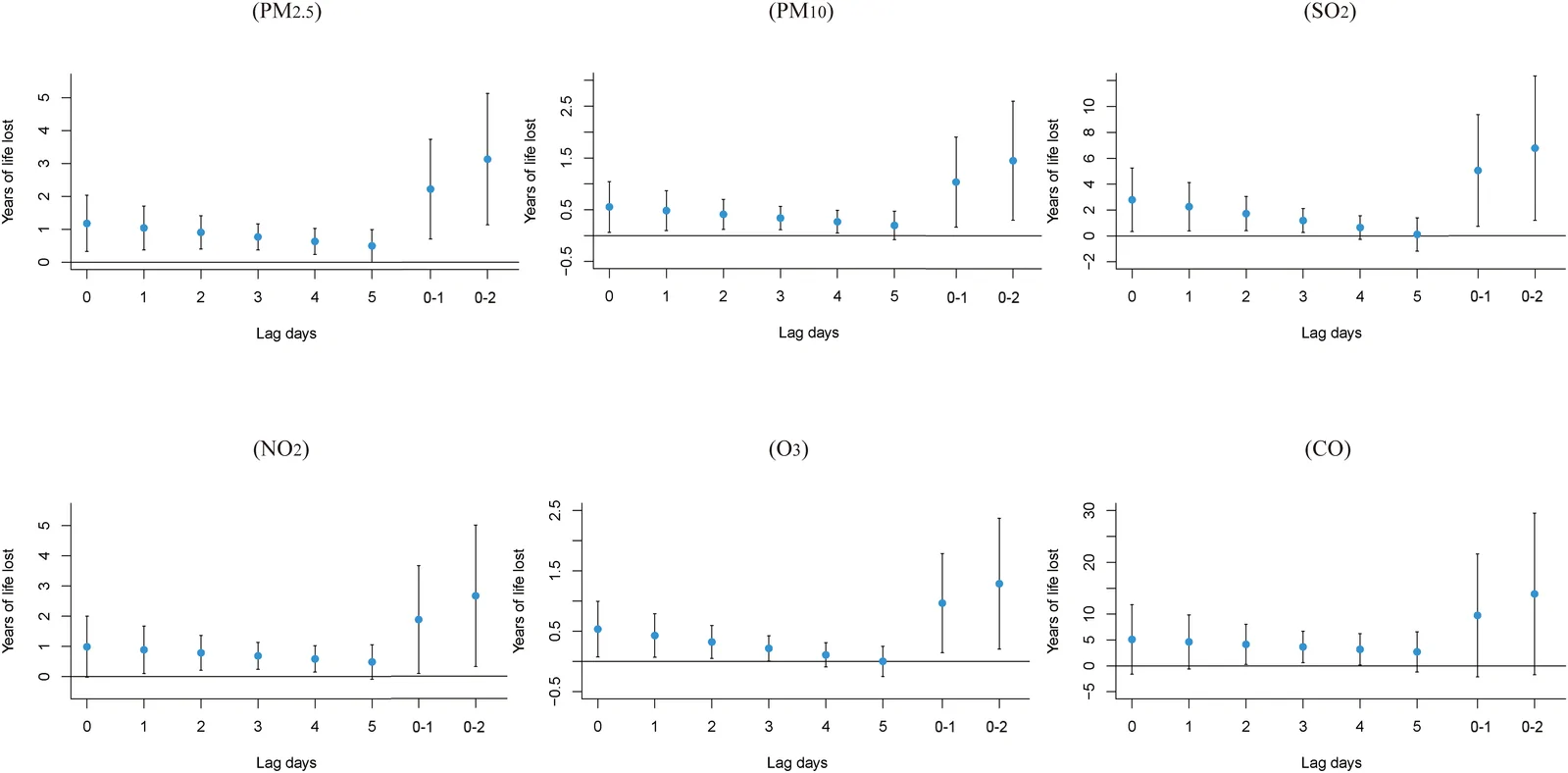
The lag-specific association between increases in air pollution exposure and YLLs from cancer using single-pollutant models in Pudong New Area, 2013-2021.

**Fig 2:**
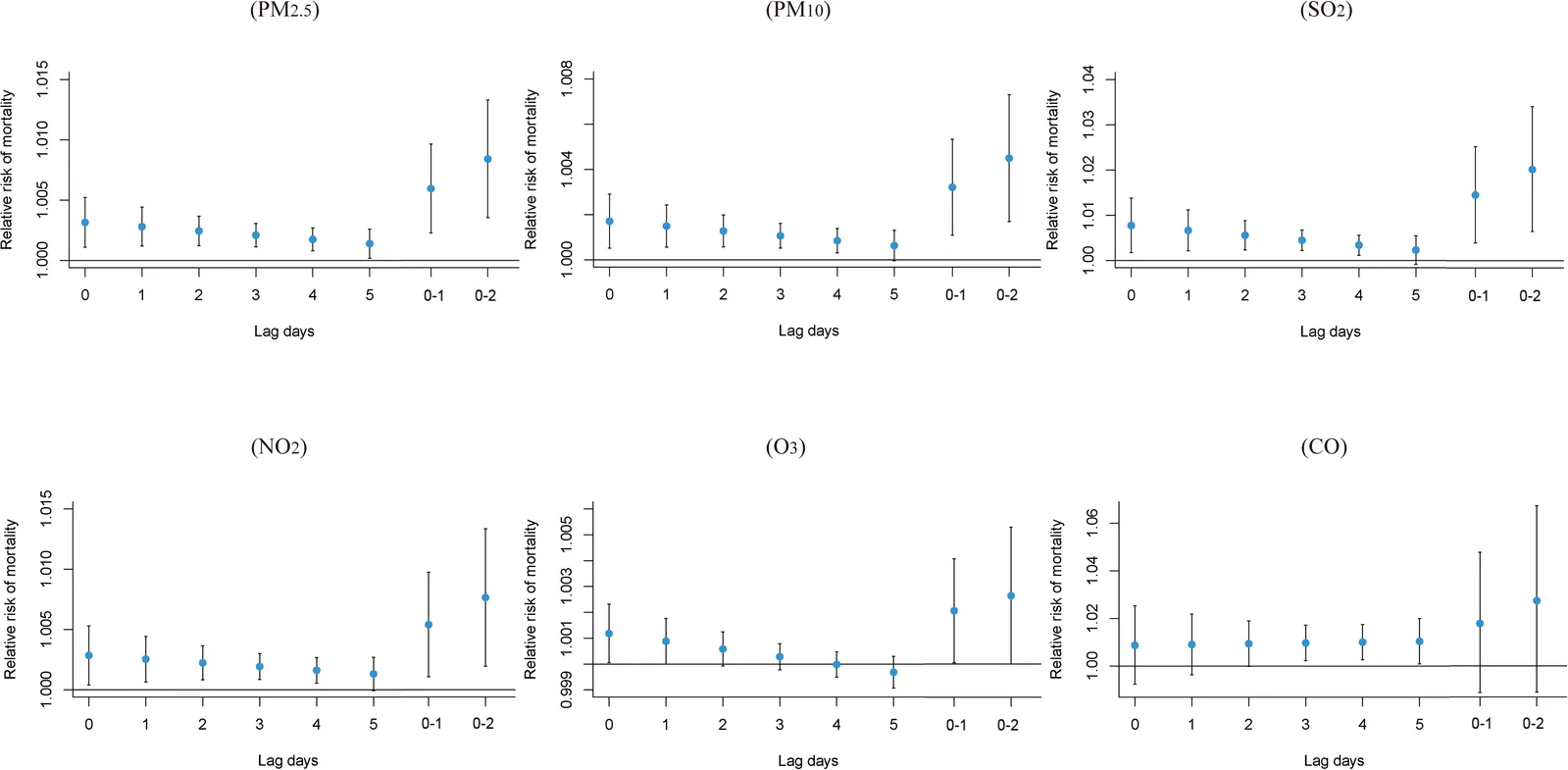
The lag-specific association between increases in air pollution exposure and RR from cancer mortality using single-pollutant models in Pudong New Area, 2013-2021.

### Estimated increases of YLLs and cancer mortality

Table 3 presents the estimated increases of YLLs and ER (%) from cancer mortality due to air pollution exposure using single- and two-pollutant models. In single-pollutant models, each 10 μg/m^3^ increase in PM_2.5_, PM_10_, SO_2_, NO_2_, and O_3_ exposure was positively associated with YLL increases of 2.22 (95% CI: 0.71, 3.74), 1.04 (95% CI: 0.17, 1.91), 5.05 (95% CI: 0.74, 9.36), 1.88 (95% CI: 0.09, 3.66), and 0.96 (95% CI: 0.14, 1.78) years on lag0-1, respectively. Moreover, each 10 μg/m^3^ increase in PM_2.5_, PM_10_, SO_2_, NO_2_, and O_3_ exposure was positively associated with ER% increases of 0.59 (95% CI: 0.23, 0.96), 0.32 (95% CI: 0.11, 0.53), 1.45 (95% CI: 0.39, 2.52), 0.54 (95% CI: 0.11, 0.97), and 0.21 (95% CI: 0.01, 0.41) on lag0-1, respectively. In two-pollutant models, the estimated association for PM_2.5_ remained stable when adjusting for other five pollutants. After adjusting for PM_2.5_ and PM_10_, the estimated impacts of SO_2_, NO_2_, and O_3_ decreased significantly, suggesting that there may be a synergistic effect between gaseous and particulate pollutants, in terms of their impact on cancer. The estimated impacts of SO_2_ and O_3_ remain relatively stable after other gaseous pollutants have been controlled.

**Table 3:** The increases of YLLs and ER (%) from cancer mortality due to increased pollutant concentrations using single- and two-pollutant models on lag0-1.

Pollutants	Years of life lost	Excess risk (%) of mortality
PM_2.5_		
Single pollutant	2.22 (0.71, 3.74)	0.59 (0.23, 0.96)
+SO_2_	2.23 (0.37, 4.09)	0.50 (0.05, 0.95)
+NO_2_	2.51 (0.74, 4.27)	0.61 (0.18, 1.04)
+O_3_	2.07 (0.52, 3.62)	0.59 (0.22, 0.97)
+CO	2.50 (0.66, 4.34)	0.74 (0.29, 1.19)
PM_10_		
Single pollutant	1.04 (0.17, 1.91)	0.32 (0.11, 0.53)
+SO_2_	0.86 (−0.16, 1.87)	0.25 (0.00, 0.49)
+NO_2_	1.03 (0.04, 2.03)	0.31 (0.07, 0.55)
+O_3_	0.94 (0.05, 1.83)	0.32 (0.10, 0.53)
+CO	0.99 (−0.02, 1.99)	0.36 (0.11, 0.60)
SO_2_		
Single pollutant	5.05 (0.74, 9.36)	1.45 (0.39, 2.52)
+PM_2.5_	3.65 (−1.54, 8.84)	1.26 (−0.01, 2.54)
+PM_10_	3.92 (−0.96, 8.79)	1.23 (0.04, 2.43)
+NO_2_	5.07 (0.16, 9.99)	1.43 (0.23, 2.65)
+O_3_	4.78 (0.44, 9.13)	1.43 (0.37, 2.51)
+CO	4.74 (−0.02, 9.50)	1.58 (0.42, 2.77)
NO_2_		
Single pollutant	1.88 (0.09, 3.66)	0.54 (0.11, 0.97)
+PM_2.5_	0.97 (−1.19, 3.13)	0.33 (−0.19, 0.85)
+PM_10_	1.17 (−0.92, 3.27)	0.35 (−0.15, 0.86)
+SO_2_	1.65 (−0.47, 3.78)	0.37 (−0.14, 0.89)
+O_3_	1.67 (−0.15, 3.50)	0.53 (0.09, 0.97)
+CO	1.80 (−0.30, 3.89)	0.60 (0.09, 1.11)
O_3_		
Single pollutant	0.96 (0.14, 1.78)	0.21 (0.01, 0.41)
+PM_2.5_	0.83 (−0.03, 1.69)	0.17 (−0.04, 0.38)
+PM_10_	0.84 (−0.01, 1.68)	0.17 (−0.04, 0.38)
+SO_2_	1.01 (0.18, 1.83)	0.21 (0.01, 0.42)
+NO_2_	1.15 (0.30, 2.00)	0.22 (0.01, 0.43)
+CO	0.97 (0.12, 1.82)	0.20 (−0.01, 0.41)
CO		
Single pollutant	9.74 (−2.15, 21.62)	1.78 (−1.12, 4.77)
+PM_2.5_	0.95 (−14.17, 16.06)	−0.96 (−4.55, 2.77)
+PM_10_	3.31 (−10.98, 17.60)	−0.41 (−3.82, 3.12)
+SO_2_	5.72 (−8.23, 19.66)	−0.25 (−3.58, 3.20)
+NO_2_	9.38 (−4.69, 23.44)	0.82 (−2.58, 4.33)
+O_3_	8.20 (−3.93, 20.34)	1.61 (−1.35, 4.65)

+ pollutant: the specified pollutant was added to the two-pollutant model.

However, the estimates of SO_2_ and CO were reduced when adjusted for PM_2.5_ or PM_10_. The estimated impact of NO_2_ was weakened in most adjusted models.

### Age, sex, and education subgroups

Table 4 illustrates the modified effects of sex, age, and education level on YLLs and ER (%) from cancer mortality using single-pollutant models. The greater impacts on YLLs and cancer mortality were observed in males, younger populations, and higher education populations. Within subgroups defined by different demographic factors, the confidence intervals for the estimated impacts of some pollutants were no longer statistically significant. In addition, the impact of O_3_ exposure on cancer mortality was statistically different between low- and high-education subgroups, indicating heterogeneity in cancer patients’ responses to ozone exposure across different educational levels in the study area.

**Table 4:** Modified effects of sex, age, and education level on YLLs and ER (%) from cancer mortality due to air pollution exposure using single-pollutant models on lag0-1.

Indicator	Male	Female	Age < 75 years	Age ≥ 75 years	Low education	High education
**YLLs**						
PM_2.5_	**1.67 (0.55, 2.79)**	0.55 (−0.46, 1.57)	**1.77 (0.36, 3.19)**	0.45 (−0.05, 0.95)	0.67 (−0.17, 1.51)	**1.55 (0.33, 2.78)**
PM_10_	**0.88 (0.24, 1.53)**	0.16 (−0.43, 0.74)	0.73 (−0.09, 1.54)	**0.31 (0.02, 0.60)**	0.34 (−0.14, 0.83)	0.70 (−0.01, 1.40)
SO_2_	**3.26 (0.07, 6.45)**	1.79 (−1.09, 4.68)	**4.13 (0.10, 8.17)**	0.92 (−0.50, 2.33)	1.72 (−0.68, 4.13)	3.33 (−0.17, 6.82)
NO_2_	**1.44 (0.12, 2.76)**	0.43 (−0.76, 1.63)	1.37 (−0.30, 3.04)	0.51 (−0.08, 1.10)	0.34 (−0.65, 1.34)	**1.53 (0.09, 2.98)**
O_3_	0.59 (−0.01, 1.20)	0.37 (−0.18, 0.92)	**0.86 (0.09, 1.63)**	0.11 (−0.16, 0.38)	0.07 (−0.39, 0.52)	**0.90 (0.23, 1.56)**
CO	8.59 (−0.20, 17.38)	1.15 (−6.80, 9.10)	9.06 (−2.06, 20.18)	0.68 (−3.23, 4.59)	2.77 (−3.85, 9.39)	6.97 (−2.66, 16.60)
**ER%**						
PM_2.5_	**0.61 (0.14, 1.07)**	0.58 (−0.02, 1.18)	**0.75 (0.25, 1.25)**	0.41 (−0.12, 0.95)	0.40 (−0.14, 0.94)	**0.78 (0.28, 1.28)**
PM_10_	**0.39 (0.12, 0.65)**	0.22 (−0.13, 0.56)	**0.32 (0.04, 0.61)**	**0.32 (0.01, 0.62)**	0.20 (−0.11, 0.51)	**0.43 (0.14, 0.72)**
SO_2_	1.18 (−0.15, 2.53)	**1.89 (0.16, 3.64)**	**1.74 (0.30, 3.19)**	1.12 (−0.39, 2.65)	1.34 (−0.18, 2.89)	**1.56 (0.11, 3.03)**
NO_2_	**0.55 (0.01, 1.10)**	0.52 (−0.18, 1.24)	0.56 (−0.02, 1.15)	0.51 (−0.11, 1.14)	0.33 (−0.30, 0.97)	**0.72 (0.14, 1.31)**
O_3_	0.20 (−0.05, 0.45)	0.21 (−0.11, 0.54)	**0.31 (0.04, 0.58)**	0.09 (−0.20, 0.38)	−0.04 (−0.34, 0.25)	0.43 (0.16, 0.70)*
CO	2.09 (−1.56, 5.88)	1.30 (−3.39, 6.21)	3.09 (−0.86, 7.20)	0.25 (−3.86, 4.53)	1.39 (−2.79, 5.75)	2.15 (−1.79, 6.24)

Significant impacts were presented in bold font; *P < 0.05, significantly different from the high-education subgroup.

## Discussion

Although past studies have suggested that long-term and short-term exposure to ambient air pollution was associated with the risk of cancer mortality [20–23], the loss of life expectancy due to premature death among cancer patients has been overlooked. In this study, YLL was selected as a supplementary indicator to emphasize the significance of premature death. It can help public health departments identify which diseases or injuries are the primary causes of the greatest potential loss of life in society.

### Impacts of air pollutants on cancer outcomes

In single-pollutant models, we found each 10 μg/m^3^ increase in PM_2.5_, PM_10_, SO_2_, NO_2_, and O_3_ exposure was significantly associated with YLL increases of 2.22 (95% CI: 0.71, 3.74), 1.04 (95% CI: 0.17, 1.91), 5.05 (95% CI: 0.74, 9.36), 1.88 (95% CI: 0.09, 3.66), and 0.96 (95% CI: 0.14, 1.78) years on lag0-1, respectively. Additionally, each 10 μg/m^3^ increase in exposure to the five pollutants was significantly associated with ER% increases of 0.59 (95% CI: 0.23, 0.96), 0.32 (95% CI: 0.11, 0.53), 1.45 (95% CI: 0.39, 2.52), 0.54 (95% CI: 0.11, 0.97), and 0.21 (95% CI: 0.01, 0.41) on lag0–1, respectively. Therefore, short-term exposure to PM_2.5_, PM_10_, SO_2_, NO_2_, and O_3_ has a significant impact on cancer-related YLLs and mortality in Pudong New Area, Shanghai. Yu and Coleman et al. have similar research findings for cancer mortality [21,24]. However, the burden of cancer-related YLLs has been rarely reported. It is important to recognize that the loss to families and society caused by the death of young people is greater than that caused by the death of the elderly.

The biological mechanisms underlying the impact of short-term exposure to outdoor air pollution on the risk of all cancer mortality remain unclear. Current researches mainly suggest that ambient air pollution contributes to the pathological process of cancer primarily through oxidative stress, immune-inflammatory responses, DNA methylation, and gene mutations [25]. Air pollutants inhaled by the body may affect areas such as the thoracic cavity, trachea, bronchi, or alveolar airways along the respiratory tract. Residual particles and gases can have significant local and systemic effects, causing low-grade and long-term inflammation and oxidative stress [26]. Short-term exposure to environmental air pollution is associated with abnormal DNA methylation [27]. In addition, Yu et al. have shown that the number of gene mutations in lung cancer associated with air pollution is three times higher than in lung cancer in low-exposure areas [28].

### Possible interactions between individual pollutants

In two-pollutant models, we found that the estimated impacts of exposure to individual air pollutants were generally attenuated after adjusting for other pollutants. This finding may suggest that various air pollutants exhibited synergistic effects on the short-term impact of cancer burden. Here are some studies that support our viewpoint. Gawelko et al. have demonstrated that NO_2_ exercises a synergistic leading effect. When NO_2_ interacts with other pollutants, it becomes more persistent in explaining the higher incidence of cancer [29]. A two-stage time series analysis in 372 cities has reported a synergistic effect of PM_2.5_ and O_3_ on total mortality [30]. Another controlled clinical trial has suggested that assessment of health effects by ambient particulate matter should consider its interactions with gaseous copollutants [31]. Therefore, it is meaningful to explore the differences in impacts between co-exposure to air pollutants and exposure to individual pollutants in the future. However, current statistical methods that can be used to roughly estimate the impacts of co-exposure to pollutants (such as WQS regression and BKMR) cannot be statistically compared with the impacts of exposure to individual pollutants [32,33].

### Modification effects of demographic factors

The subgroup analysis has shown that the greater impacts due to air pollution exposure on YLLs and cancer mortality were observed in male cancer patients. Similar to our research findings, Wang et al. have also found that male lung cancer patients were more sensitive to the short-term impact of air pollution exposure [14]. The findings of Giannoula et al. and Zha et al. have supported that male cancer patients constituted a susceptible population to air pollution exposure [34,35]. However, an ecological study has revealed a significant association between increased cancer mortality and air pollution exposure was merely observed in female populations [7]. One possible reason for our viewpoints is that the prevalence of smoking and alcohol abuse among men is generally higher than among women in China [36,37]. Tobacco and alcohol are associated with multiple types of cancer, including lung cancer, throat cancer, esophageal cancer, oral cancer, liver cancer, and colorectal cancer [38,39]. For another, men are more likely to work in industrial occupations such as mining, construction, and metallurgy, exposing them to chemical carcinogens like asbestos, diesel exhaust, and heavy metals [40].

In addition, the short-term impact of ambient air pollution on YLLs and cancer mortality was greater among younger populations than among older populations. Consistent with our findings, a case crossover study in Brazil has also reported that the short-term impacts of PM_2.5_ exposure on lung cancer mortality were greater in subgroups with age < 65 year [41]. A possible explanation is that young people spend more time outdoors than older adults due to better physical health, greater social opportunities, and increased work demands. However, the viewpoint that older adults face a higher risk of cancer mortality when exposed to ambient air pollution has been widely recognized [14,42,43]. Compared to younger people, older adults typically have poorer health, accompanied by various chronic diseases and complications, reduced immune function, and diminished self-repair capabilities [44]. Convincing research is needed to explore the heterogeneity in the impact of air pollution exposure on cancer mortality among age-stratified subgroups.

Finally, stronger associations between air pollution exposure and cancer outcome indicators were found in subgroups with higher education. Inconsistent with our research results, Kan et al. and Cheng et al. have revealed that the impacts of air pollution exposure were generally greater in residents with low education [7,45]. One possible explanation for our findings is that populations with higher education place greater emphasis on healthy lifestyles, including regular outdoor physical activities such as running and cycling. These activities often take place near roads, exposing participants to vehicle exhaust fumes while breathing intensely during exercise [46]. For another, they typically have better access to healthcare and heightened health awareness. They undergo regular medical check-ups and seek timely medical attention, resulting in more accurate cancer diagnoses and mortality records. Additionally, the difference in the impact of O_3_ exposure on cancer mortality among low- and high-education subgroups was statistically significant, suggesting heterogeneity in cancer patients’ responses to ozone exposure across different educational levels in the study area.

### Shortcomings

This study has the following limitations. First, replacing individual exposure levels with pollutant monitoring data from a fixed monitoring site inevitably resulted in measurement errors for each individual. This type of error can be classified as a non-differential misclassification, which typically suggested that the significant association we reported is likely a conservative underestimation of the true health risk, rather than an overestimation. Second, the data collected in this study are limited to Pudong New Area, Shanghai, so caution is required when generalizing the findings to other regions. Third, as an ecological study, we cannot draw causal conclusions but rather present the associations between air pollution exposure and cancer outcomes at the population level.

## Conclusions

In summary, we found that short-term exposure to ambient air pollution was positively associated with increases in YLLs and cancer mortality in Pudong New Area, Shanghai. Marginal differences in impact estimates were observed among men, younger populations, and those with higher education. There is heterogeneity in the response to O_3_ exposure among cancer patients across different educational levels in the study area. YLL serves as an indispensable strategic assessment tool in modern public health, guiding society to focus not merely on reducing deaths, but on extending healthy lives.

## Supporting information

S1 Fig Kernel density plot of daily years of life lost from cancer in Pudong New Area, Shanghai from 2013 to 2021. (TIF)

S1 Table Life expectancy of male population in Pudong New Area of Shanghai, 2013–2021. (PDF)

S2 Table Life expectancy of female population in Pudong New Area of Shanghai, 2013–2021. (PDF)

S3 Table Associations of per 10 μg/m^3^ increase in PM_2.5_ with years of life lost and excess risk from cancer when parameters in the models were changed. The unit of years of life lost and excess risk is year and percentage (%), respectively. The models for PM_2.5_ were based on lag0-1.
(PDF)

S4 Table Associations of per 10 μg/m^3^ increase in PM_10_ with years of life lost and excess risk from cancer when parameters in the models were changed. (PDF)

S5 Table Associations of per 10 μg/m^3^ increase in SO_2_ with years of life lost and excess risk from cancer when parameters in the models were changed. (PDF)

S6 Table Associations of per 10 μg/m^3^ increase in NO2 with years of life lost and excess risk from cancer when parameters in the models were changed. (PDF)

S7 Table Associations of per 10 μg/m^3^ increase in O_3_ with years of life lost and excess risk from cancer when parameters in the models were changed. (PDF)

S8 Table Associations of per 10 μg/m^3^ increase in CO with years of life lost and excess risk from cancer when parameters in the models were changed. (PDF)

S9 File Research data. (XLSX)
