# Supplementary material for: The short-term impact of ambient air pollution on years of life lost from cancer in Pudong New Area, Shanghai: A time series study
Source: PLoS One. 2026 Oct 27;21(10):e0360049. doi: 10.1371/journal.pone.0360049 (PMC13712746; doi:10.1371/journal.pone.0360049)
Supplement: Supplementary file 1 [file pone.0360049.s002.pdf]

Table S1: Life expectancy of male population in Pudong New Area of Shanghai, 2013-2021

Age group	Life expectancy in male population								
(years)	2013	2014	2015	2016	2017	2018	2019	2020	2021
<1	80.65	81.02	80.85	81.51	81.49	81.89	82.00	82.58	82.45
1-	79.93	80.27	80.06	80.68	80.75	81.06	81.11	81.67	81.55
5-	76.00	76.31	76.13	76.73	76.79	77.11	77.13	77.70	77.56
10-	71.08	71.35	71.15	71.78	71.82	72.15	72.14	72.72	72.58
15-	66.12	66.46	66.20	66.82	66.88	67.18	67.20	67.79	67.61
20-	61.23	61.52	61.28	61.91	61.95	62.24	62.26	62.85	62.70
25-	56.31	56.59	56.37	56.99	57.05	57.32	57.30	57.90	57.78
30-	51.40	51.66	51.48	52.08	52.16	52.39	52.41	52.97	52.86
35-	46.49	46.77	46.61	47.19	47.27	47.50	47.49	48.05	47.95
40-	41.67	41.92	41.75	42.31	42.40	42.62	42.61	43.18	43.07
45-	36.90	37.16	36.98	37.49	37.57	37.82	37.82	38.35	38.26
50-	32.31	32.50	32.31	32.79	32.90	33.11	33.12	33.65	33.55
55-	27.79	28.02	27.77	28.25	28.31	28.55	28.52	29.04	28.93
60-	23.48	23.67	23.43	23.99	23.95	24.24	24.18	24.70	24.45
65-	19.27	19.49	19.23	19.77	19.79	20.08	20.00	20.54	20.28
70-	15.37	15.52	15.33	15.85	15.84	16.11	16.02	16.56	16.30
75-	11.88	11.91	11.75	12.19	12.21	12.45	12.35	12.86	12.61
80-	8.96	8.98	8.74	9.11	9.07	9.38	9.18	9.76	9.41
≥85	6.63	6.66	6.39	6.85	6.69	7.02	6.83	7.42	6.92
