# Supplementary material for: The short-term impact of ambient air pollution on years of life lost from cancer in Pudong New Area, Shanghai: A time series study
Source: PLoS One. 2026 Oct 27;21(10):e0360049. doi: 10.1371/journal.pone.0360049 (PMC13712746; doi:10.1371/journal.pone.0360049)
Supplement: Supplementary file 2 [file pone.0360049.s003.pdf]

Table S2: Life expectancy of female population in Pudong New Area of Shanghai, 2013-2021

Age group	Life expectancy in female population								
(years)	2013	2014	2015	2016	2017	2018	2019	2020	2021
<1	85.48	85.65	85.57	86.07	86.36	86.74	87.02	87.01	87.07
1-	84.70	84.96	84.76	85.22	85.49	85.90	86.17	86.21	86.20
5-	80.71	81.03	80.79	81.27	81.57	81.93	82.20	82.25	82.22
10-	75.75	76.05	75.81	76.33	76.58	76.96	77.22	77.27	77.22
15-	70.79	71.10	70.87	71.38	71.63	72.03	72.28	72.33	72.28
20-	65.84	66.16	65.94	66.45	66.65	67.07	67.32	67.42	67.32
25-	60.88	61.20	61.00	61.51	61.70	62.13	62.36	62.47	62.39
30-	55.95	56.23	56.07	56.57	56.74	57.20	57.40	57.52	57.44
35-	51.01	51.29	51.15	51.63	51.82	52.27	52.44	52.58	52.48
40-	46.11	46.41	46.23	46.72	46.92	47.38	47.54	47.68	47.57
45-	41.22	41.57	41.36	41.89	42.04	42.53	42.66	42.80	42.67
50-	36.43	36.79	36.52	37.07	37.22	37.74	37.82	37.96	37.82
55-	31.70	32.02	31.79	32.33	32.49	32.98	33.07	33.20	33.09
60-	27.02	27.31	27.15	27.68	27.82	28.30	28.40	28.49	28.38
65-	22.49	22.74	22.60	23.15	23.23	23.74	23.81	23.91	23.78
70-	18.12	18.34	18.21	18.73	18.80	19.30	19.31	19.45	19.25
75-	14.08	14.32	14.13	14.56	14.72	15.12	15.10	15.26	15.07
80-	10.56	10.83	10.68	10.93	11.03	11.41	11.32	11.45	11.26
≥85	7.91	8.09	8.00	8.12	8.17	8.56	8.39	8.57	8.28
