# Supplementary material for: The short-term impact of ambient air pollution on years of life lost from cancer in Pudong New Area, Shanghai: A time series study
Source: PLoS One. 2026 Oct 27;21(10):e0360049. doi: 10.1371/journal.pone.0360049 (PMC13712746; doi:10.1371/journal.pone.0360049)
Supplement: Supplementary file 3 [file pone.0360049.s004.pdf]

Table S3: Associations of per 10 μg/m³ increase in $PM_{2.5}$ with years of life lost and excess risk from cancer when parameters in the models were changed.

Parameter changed in the model	Years of life lost (95%CI)	Excess risk (95%CI)
Original model	2.22 (0.71, 3.74)	0.59 (0.23, 0.96)
Relative humidity (4 degrees of freedom)	2.24 (0.72, 3.75)	0.60 (0.23, 0.97)
Temperature (4 degrees of freedom)	2.21 (0.69, 3.72)	0.59 (0.22, 0.96)
Time per year (1 degrees of freedom)	2.50 (1.01, 3.99)	0.71 (0.35, 1.08)
Time per year (2 degrees of freedom)	2.22 (0.71, 3.74)	0.59 (0.23, 0.96)
Time per year (3 degrees of freedom)	2.05 (0.52, 3.57)	0.55 (0.18, 0.92)
Research data from before the COVID-19 pandemic	2.45 (0.87, 4.03)	0.62 (0.24, 1.01)

The unit of years of life lost and excess risk is year and percentage (%), respectively. The models for $PM_{2.5}$ were based on lag0-1.
