# Supplementary material for: The short-term impact of ambient air pollution on years of life lost from cancer in Pudong New Area, Shanghai: A time series study
Source: PLoS One. 2026 Oct 27;21(10):e0360049. doi: 10.1371/journal.pone.0360049 (PMC13712746; doi:10.1371/journal.pone.0360049)
Supplement: Supplementary file 7 [file pone.0360049.s008.pdf]

Table S7: Associations of per 10 μg/m³ increase in O_3 with years of life lost and excess risk from cancer when parameters in the models were changed.

Parameter changed in the model	Years of life lost (95%CI)	Excess risk (95%CI)
Original model	0.96 (0.14, 1.78)	0.21 (0.01, 0.41)
Relative humidity (4 degrees of freedom)	0.97 (0.15, 1.79)	0.21 (0.01, 0.41)
Temperature (4 degrees of freedom)	0.87 (0.06, 1.68)	0.19 (-0.01, 0.39)
Time per year (1 degrees of freedom)	0.82 (0.01, 1.62)	0.15 (-0.05, 0.35)
Time per year (2 degrees of freedom)	0.96 (0.14, 1.78)	0.21 (0.01, 0.41)
Time per year (3 degrees of freedom)	0.91 (0.05, 1.76)	0.19 (-0.02, 0.40)
Research data from before the COVID-19 pandemic	1.15 (0.24, 2.05)	0.26 (0.04, 0.49)

The unit of years of life lost and excess risk is year and percentage (%), respectively. The models for O_3 were based on lag0-1.
