# Supplementary material for: The short-term impact of ambient air pollution on years of life lost from cancer in Pudong New Area, Shanghai: A time series study
Source: PLoS One. 2026 Oct 27;21(10):e0360049. doi: 10.1371/journal.pone.0360049 (PMC13712746; doi:10.1371/journal.pone.0360049)
Supplement: Supplementary file 8 [file pone.0360049.s009.pdf]

Table S8: Associations of per 10 μg/m³ increase in CO with years of life lost and excess risk from cancer when parameters in the models were changed.

Parameter changed in the model	Years of life lost (95%CI)	Excess risk (95%CI)
Original model	9.74 (-2.15, 21.62)	1.78 (-1.12, 4.77)
Relative humidity (4 degrees of freedom)	9.83 (-2.07, 21.72)	1.80 (-1.11, 4.79)
Temperature (4 degrees of freedom)	9.80 (-2.08, 21.68)	1.77 (-1.13, 4.76)
Time per year (1 degrees of freedom)	12.59 (0.85, 24.33)	3.05 (0.14, 6.04)
Time per year (2 degrees of freedom)	9.74 (-2.15, 21.62)	1.78 (-1.12, 4.77)
Time per year (3 degrees of freedom)	9.09 (-2.90, 21.09)	1.64 (-1.28, 4.65)
Research data from before the COVID-19 pandemic	14.43 (1.72, 27.15)	3.04 (-0.09, 6.28)

The unit of years of life lost and excess risk is year and percentage (%), respectively. The models for CO were based on lag0-1.
